# Association between Diabetes and Keratoconus: A Retrospective Analysis

**DOI:** 10.1038/s41598-019-50095-2

**Published:** 2019-09-24

**Authors:** Amy E. Whelchel, Tina B. McKay, Shrestha Priyadarsini, Tyler Rowsey, Dimitrios Karamichos

**Affiliations:** 1 Department of Ophthalmology/Dean McGee Eye Institute, University of Oklahoma Health Sciences Center, Oklahoma City, OK 73104 USA; 2 Department of Cell Biology, University of Oklahoma Health Sciences Center, Oklahoma City, OK 73104 USA; 3 Department of Physiology, University of Oklahoma Health Sciences Center, Oklahoma City, OK 73104 USA

**Keywords:** Corneal diseases, Vision disorders

## Abstract

Keratoconus (KC) and chronic diabetes mellitus (DM) are both associated with significant defects in the human corneal structure. Studies have long suggested that DM is linked to KC, mainly via the crosslinking mechanism, but scientific evidences are lacking. The role of altered systemic metabolism is well-established in both DM and KC with studies suggesting localized altered cellular metabolism leading to the development of corneal pathologies. We have previously characterized the metabolic defects associated with both conditions using targeted metabolomics. To compare metabolic differences between KC and DM-derived corneal fibroblasts, we performed a respective study of two cohorts of the KC and DM populations using a retrospective analysis of targeted metabolomics data. The goal of this study was to identify the group of differentially regulated metabolites, in KC versus DM, so that we may unravel the link between the two devastating corneal pathologies.

## Introduction

Keratoconus (KC) is one of the most common corneal dystrophies and is known to affect 1/400-1/2000 persons worldwide. KC is a progressive disease known to cause irregular corneal astigmatism, in early stages, and corneal thinning with ectasia, in more advanced disease. Clinically, vision correction for KC patients is provided by spectacles, rigid gas permeable contact lenses, and penetrating keratopathy, depending on the stage of the disease. Collagen crosslinking (CXL) is one of the newest treatments for KC, for those who qualify. Riboflavin (Vitamin B2) and ultraviolet A light are applied together to strengthen the collagen bonds in corneal extracellular matrix (ECM) and stabilize the corneal tissue by forming additional covalent bonds between collagen fibrils and proteoglycan core proteins^1^. CXL is widely considered successful, although the data is rather limited in United States (the FDA approved CXL in 2016). Patients with diabetes mellitus (DM) also experience strengthening of their corneal stroma characterized by increased crosslinking between collagen fibrils^2–4^, only this process is related to aging and the prolonged hyperglycemic state. Epidemiological studies have suggested that DM patients have roughly 20–52% lower odds of KC development depending on DM severity compared to healthy controls^5^. It has been, therefore, postulated that patients with DM are somewhat protected against the development of KC^6,7^. It is definitely an intriguing hypothesis that remains understudied, with very few studies reported.

The role of cellular metabolism in regulating cell differentiation, proliferation, and death has been well characterized in different tissue and disease models, including cancer^8,9^ and obesity^10^. The means by which a cell responds to extracellular stimuli or stress, *e.g*. nutrient deprivation, hypoxia, UV-light, is influenced by epigenetic regulators of gene expression and metabolic flux in response to changes in the microenvironment^11,12^. The cornea is particularly susceptible to these environmental stresses as an external, avascular tissue. Both KC and DM have been associated with increased oxidative stress or hypersensitivity to oxidation within the cornea^13–19^. We have previously identified an increase in lactate production corresponding to upregulation in aerobic glycolysis in primary corneal stromal cells isolated from KC patients, termed HKCs, compared to healthy controls (HCFs)^16,20^. Furthermore, we identified that inhibition of lactate production using the small molecule, Quercetin, decreased expression of the fibrotic markers, α-smooth muscle actin (α-SMA) and Collagen III, suggesting that changes in glucose metabolism may influence ECM deposition^20,21^. Studies of the metabolic changes in stromal keratocytes isolated from DM patients have likewise shown variations in basal cellular metabolism corresponding to increased Collagen III production^22^, though heavily favoring alternative pathways including tryptophan metabolism depending on Type 1 DM (T1DM) or Type 2 DM (T2DM)^23^. This work has led us to hypothesize that differential metabolic regulation may be a key factor in promoting the pathological changes in corneal structure, *e.g*. thinning and thickening, in the context of KC development and DM progression, respectively.

The aim of the current study was to draw parallel and contrasting features of the metabolic network of both KC- and DM-derived corneal stromal cells as a means to determine key characteristics in bioenergetics that contribute to pathological changes in corneal structure associated with each disease. In order to do that, we performed a retrospective analysis of multiple targeted metabolomics datasets with a cross comparison of the metabolome of corneal fibroblasts isolated from healthy controls, KC-, T1DM-, and T2DM-patients. These studies utilized our established 3D *in vitro* model where primary cells are stimulated by stable ascorbic acid (Vitamin C) to assemble their own ECM. Using targeted mass spectrometry, we retrospectively performed a higher-order analysis of regulated metabolic pathways by comparing the relative abundance of 134 metabolites in order to determine similarities and differences between the DM and KC samples.

Metabolomics has been used, by us and others^20,21,23–26^, for the investigation of ocular diseases. However, it has not been utilized to its full potential in terms of identifying therapeutic targets for diseases. To our knowledge, this is the first study attempting to shine a light on the corneal metabolic associations between KC and DM.

## Materials and Methods

### Ethical approval and informed consent

The study was performed with the Institutional Review Board (IRB) approval from the University of Oklahoma Health Sciences Center (IRB protocol #3450). Written, informed consent was obtained prior to tissue collection. All methods were performed in accordance with federal and institutional guidelines. All human samples were de-identified prior to analysis. This research adhered to the tenets of the Declaration of Helsinki.

### Study rationale and objectives

A retrospective analysis of previously reported metabolomics studies of Healthy, KC, T1DM, and T2DM corneal stromal cells, in our 3D *in vitro* model, was performed using a cross-comparison analysis of multiple experiments^22,27^. These metabolic experiments have previously highlighted dysregulated metabolic pathways or drug-regulated pathways (*e.g*. prolactin) related to each disease compared to healthy controls. The objectives of the current study included the following: i) identify differential regulation of metabolic pathways that may be shared (up- or down-regulated) between KC and DM; and ii) directly compare similarities and differences in metabolism, in KC and DM, that may contribute to corneal crosslinking.

### Data compilation

Targeted metabolomics data was collected and processed at the Mass Spectrometry Core at Beth Israel Deaconess Medical Center. All samples were isolated and analyzed using identical protocols, as previously described^16,21–23^. Two cohorts of each population (healthy, KC, and DM)^22,27^ were chosen with no added chemicals or treatments, as used in other metabolomics studies previously reported by our lab^21^.

### Inclusion and exclusion criteria

Only metabolites identified in all biological replicates in each group were included in the analysis. Two independent datasets^22,27^ were evaluated using HCFs obtained from healthy controls, HKCs isolated from KC patients, and T1DM and T2DM isolated from DM patients. Any metabolites with no expression detected in a biological replicate were excluded from further analysis.

### Cell isolation and expansion

Human corneal stromal cells were isolated from healthy, KC, T1DM, and T2DM patients. All healthy samples, with no ocular or systemic diseases, were obtained from NDRI (National Disease Research Interchange; Philadelphia, PA). All KC samples were obtained from Aarhus University Hospital (Aarhus, Denmark) and our collaborator Dr. Jesper Hjortdal. The average age range for HCF and HKC donors reported for one independent data set^27^ was 46 ± 22 and 47 ± 14 years old. T1DM and T2DM samples were obtained from the Oklahoma Lions Eye Bank (Oklahoma City, OK), as well as the NDRI. The average age range for HCF and T1DM and T2DM donors for the second data set^22^ were 58 ± 6, 55 ± 7, and 59 ± 5 years old, respectively^22^. An average duration of DM was reported as 15.71 ± 4.17 years with a range of 3–30 years^22^. Tissue was processed, as previously described^28^. Briefly, the corneal epithelium and the corneal endothelium were removed from the stroma by scraping with a razor blade. The stromal tissue was then cut (~2 × 2 mm pieces) and positioned into T25 culture flasks. Explants were allowed to adhere and cultured with Eagle’s Minimum Essential Medium (EMEM: ATCC; Manassas, VA), 10% fetal bovine serum (FBS: Atlantic Biologicals; Miami, FL) and 1X antibiotic-antimycotic (Gibco, Life Technologies; Grand Island, NY). All cells used for our experiments were between passages 3 and 7.

### 3D *in vitro* constructs

As previously described^20–22^, HCFs, HKCs, T1DMs, and T2DMs were plated on 6-well transwell polycarbonate membrane inserts with 0.4 μm pores (Corning Costar; Charlotte, NC) at a density of 10^6^ cells/well. All cells were cultured in EMEM with 10% FBS and 1X antibiotic-antimycotic and were further stimulated with 0.5 mM 2-O-α-D glucopyranosyl-L-ascorbic acid (American Custom Chemicals Corporation, San Diego, CA, USA). The cultures were allowed to grow for 4 weeks before processing.

### Isolation, extraction of metabolites and targeted mass spectrometry

#### Sample preparation and processing

All constructs consisting of HCFs, HKCs, T1DMs, and T2DMs were processed and metabolites were isolated as previously described^20–22^. Briefly, samples were washed with 1xPBS and lysed with ice-cold 80% methanol, incubated on dry ice for 15 minutes, and homogenized briefly to ensure complete cell lysis. Samples were then centrifuged at 13,500 rpm overnight at 4 °C and stored at −80 °C until further analysis. Pellets were re-suspended in 20 μL HPLC-graded water for targeted tandem mass spectrometry.

#### Mass spectrometry processing

Briefly, 5 μL of each sample was injected and analyzed using a hybrid 5500 QTRAP triple quadrupole mass spectrometer (AB/SCIEX) coupled to a Prominence UFLC system (Shimadzu) using an Amide HILIC column (Waters) and analyzed with selected reaction monitoring (SRM) with positive/negative polarity switching. Peak areas from the total ion current for each of 134 metabolite SRM transition were integrated using MultiQuant v2.1 software (AB/SCIEX). Relative metabolite abundance was provided as integrated peak intensities.

#### Data analysis

Retrospective data, from previous studies^22,27^, was analyzed and plotted. Each of these experiments was performed from two independent studies using at least 3 biological replicates per group (HCFs, HKCs, T1DMs, and T2DMs). The data for each test group (HKC, T1DM, and T2DM) were first normalized relative to their respective experimental control (HCF) allowing for comparability between groups and experiments based on the up-/down-regulation relative to control. The original raw data from the MultiQuant software was uploaded to MetaboAnalyst (http://www.metaboanalyst.ca) for subsequent data processing and analyses^29,30^. Over Representation Analysis (ORA) was performed using only the metabolites that are regulated by 2:1 ratio, as previously described^16^, in order to ensure that only the vastly abundant metabolites were included.

### Pathway Enrichment

We performed Pathway Enrichment Analysis using a Metaboanalyst (www.Metaboanalyst.ca), which is intended for the analysis of metabolomics data^29^. As previously reported, only the metabolites that were up or down regulated by 2-fold were included in the analysis. The 2-fold cutoff ensures that only the vastly abundant metabolites were included. Furthermore, only metabolites that were detected in all biological samples were included. The metabolites fulfilling our criteria were input into the software and the pathway enrichment analysis was executed. The output of this algorithm highlights metabolic pathway or pathways affected.

### Statistical analysis

Data was generated from our previous metabolic studies on diabetes in the cornea and keratoconus separately^22,27^ and was compiled for this retrospective analysis. All data was plotted with at least an n ≥3 with statistical significance determined using a one-way ANOVA with Tukey’s multiple comparison test. A p-value of less than 0.05 was considered statistically significant.

## Results

### Altered Metabolic Pathways

**Figure 1:**
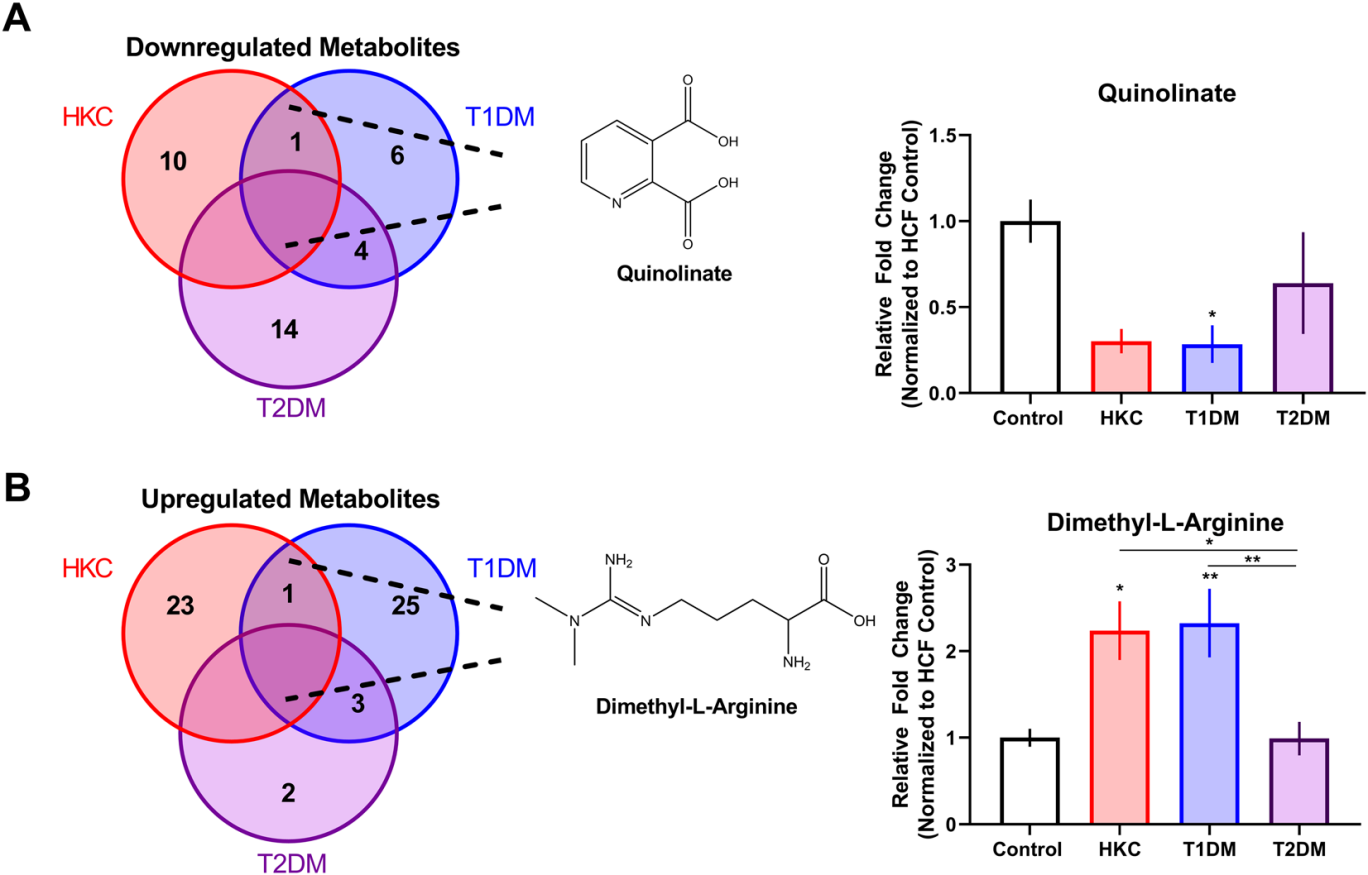
Shared metabolites in HKCs, T1DM, and T2DM constructs with down- and up-regulation compared to healthy controls. These numbers only represent the metabolites with a 2-fold change or higher. (**A**) Total number of metabolites downregulated in diseased corneal fibroblasts compared to healthy controls. The only metabolite downregulated in only the HKCs and T1DM cells is quinolinate, a metabolite associated as an agonist of the glutamate receptor (NMDA). (**B**) Total number of metabolites upregulated in diseased corneal fibroblasts compared to healthy controls. The only metabolite upregulated in both HKCs and T1DM cells is dimethyl-L-arginine, an endogenous inhibitor of nitric oxide synthase. All cells utilized in this study are human-derived corneal fibroblasts. Error bars represent standard error of the mean based on n = 3–7. *p ≤ 0.05 and **p ≤ 0.01 based on a one-way ANOVA using Tukey’s multiple comparison test.

A retrospective analysis of the metabolomics data indicated that out of 134 targeted metabolites, 11, 11, and 18 metabolites were downregulated at least 2-fold in HKCs, T1DM, and T2DM cells, respectively, compared to HCFs (Fig. 1A). Quinolinate, an agonist of the glutamate receptor, was the only metabolite downregulated in both KC and T1DM (p ≤ 0.05) and not in the T2DM. Both HKCs and T1DM cells exhibited significant upregulation of 24 and 29 metabolites relative to the HCFs, respectively, compared to only 5 metabolites upregulated in T2DM (Fig. 1B). Interestingly, dimethyl-L-arginine, an inhibitor of nitric oxide synthase, was the only metabolite shared in a 2-fold upregulation in HKCs (p ≤ 0.05) and T1DM (p ≤ 0.01) compared to HCFs, with no significant variation found in T2DM.

**Figure 2:**
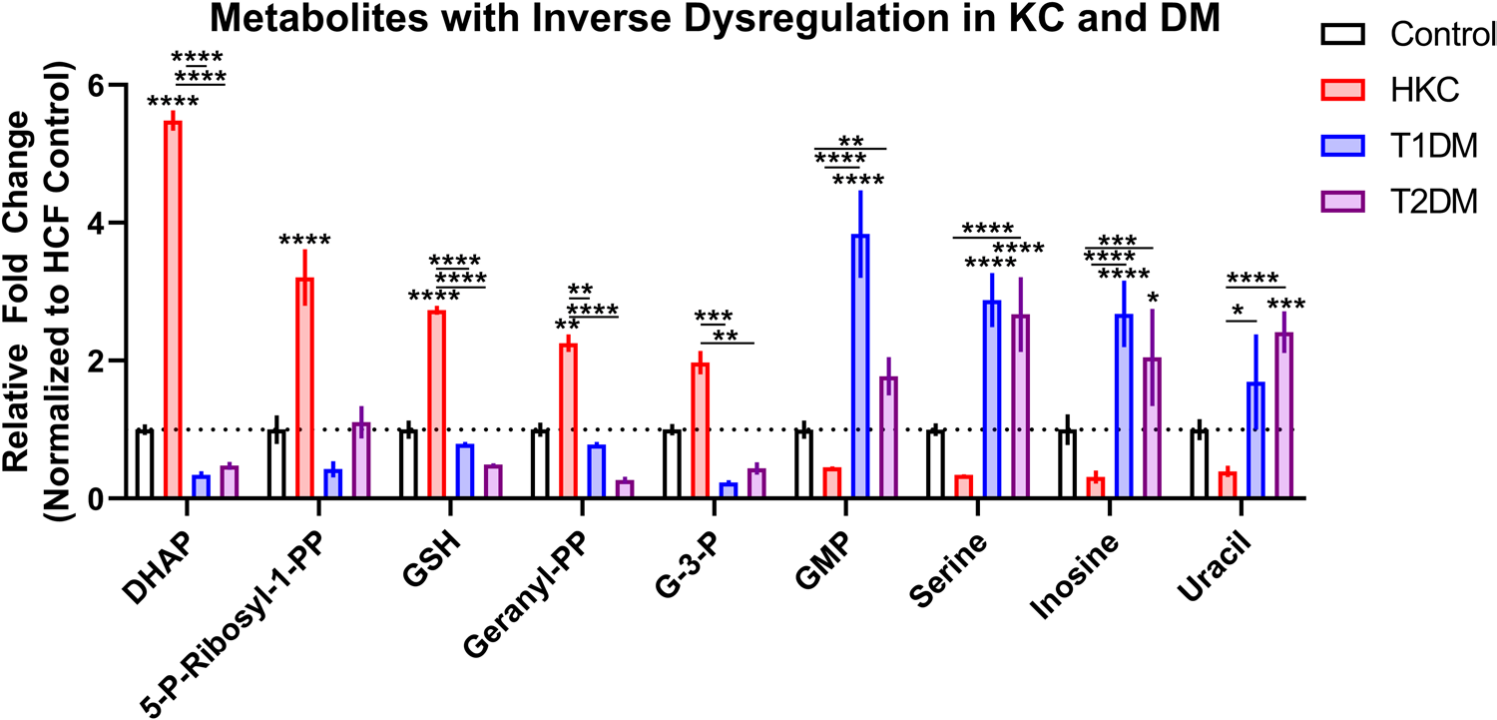
Differential metabolite levels in KC and DM constructs based on a 2-fold increase or decrease relative to healthy controls (HCFs). All cells utilized in this study are human-derived corneal fibroblasts. Error bars represent standard error of the mean based on n = 3–7. *p ≤ 0.05, **p ≤ 0.01, ***p ≤ 0.001, and ****p ≤ 0.0001 based on a one-way ANOVA using Tukey’s multiple comparison test. Dotted line denotes the relative location of the healthy control set to 1. (Abbreviations: dihydroxyacetone phosphate (DHAP), 5-phospho-ribosyl-1-pyrophosphate (5-P-Ribosyl-1-PP), glutathione (GSH), geranyl pyrophosphate (geranyl-PP), glyceraldehyde-3-phosphate (G-3-P), and guanosine monophosphate (GMP)).

Our analysis indicated that 9 metabolites were significantly different between HKCs and T1DM/T2DM with at least a 2-fold differential from healthy controls, as seen in Fig. 2. Interestingly, these 9 metabolites were inversely regulated in HKCs compared to their DM counterparts, with upregulation of glycolytic intermediates, *e.g*. dihydroxyacetone phosphate and glyceraldehyde-3-phosphate, by 5.5-fold and 2-fold (p ≤ 0.0001), respectively. In contrast, dihydroxyacetone phosphate and glyceraldehyde-3-phosphate were downregulated by 2.9-fold and 2.1-fold in T1DMs and T2DMs, respectively (Fig. 2). This trend of differential upregulation in HKCs and downregulation in DMs includes other metabolites, such as glutathione, 5-phospho-ribosyl-1-pyrophosphate, and geranyl-pyrophosphate. Metabolites that were upregulated in DM constructs but downregulated in HKCs, compared to controls, included nucleic acid derivatives, *e.g*. guanosine monophosphate (3.8-fold in T1DM, p ≤ 0.0001), inosine (2-fold in T1DM and T2DM, p ≤ 0.001), and uracil (2.4-fold in T2DM, p ≤ 0.0001), and the amino acid serine (2-fold in T1DM and T2DM, p ≤ 0.0001) (Fig. 2).

**Figure 3:**
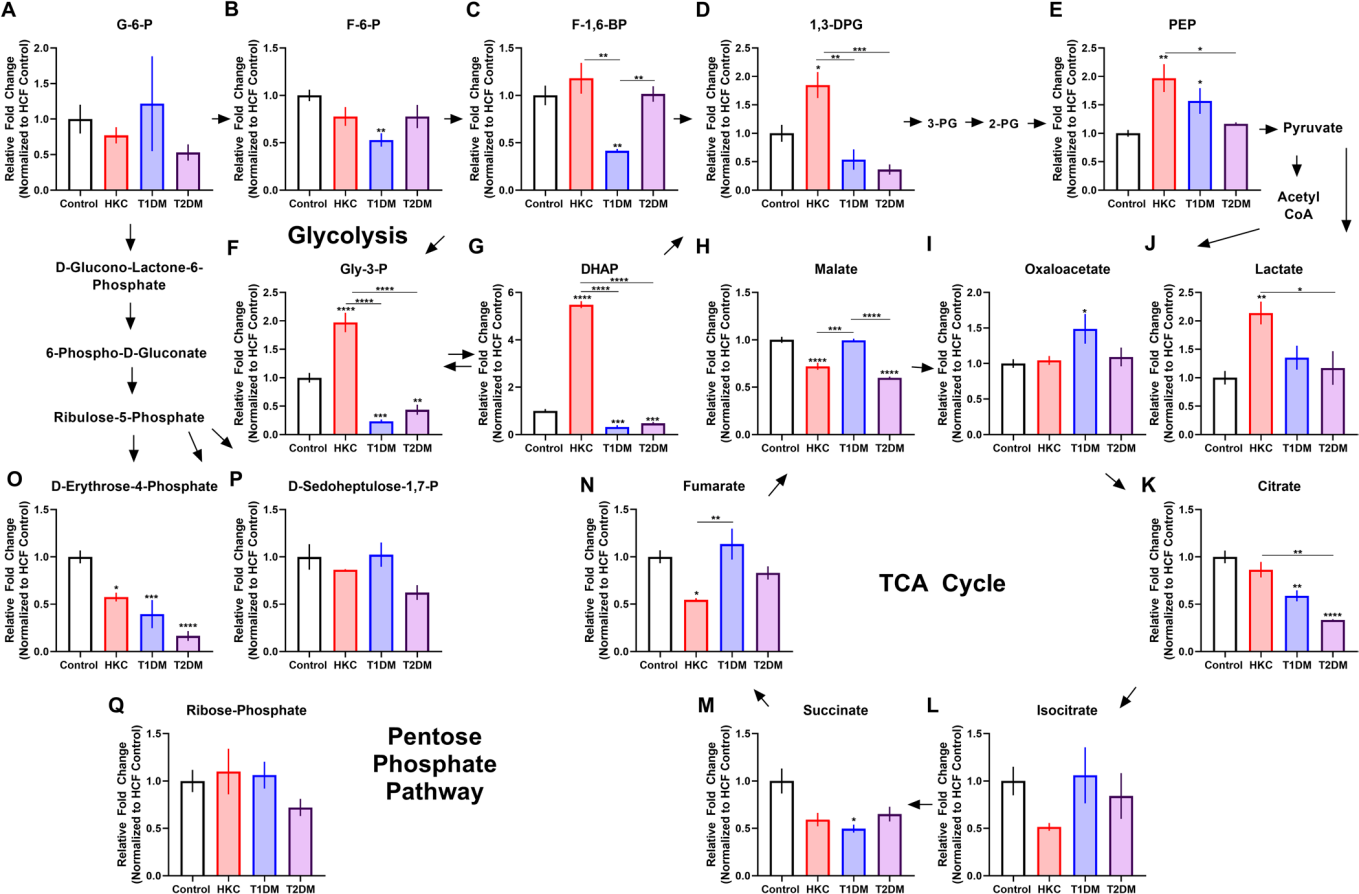
Comparison of glucose metabolism in HKCs, T1DM, and T2DM constructs. Steady-state metabolite levels involved in (**A**–**G**) glycolysis, (**H,I**, **K**–**N**) tricarboxylic acid (TCA) cycle, (**J**) lactate production, and (O-Q) pentose phosphate pathway. All cells utilized in this study are human-derived corneal fibroblasts. Error bars represent standard error with *p ≤ 0.05, **p ≤ 0.01, ***p ≤ 0.001, and ****p ≤ 0.0001 based on a one-way ANOVA with Tukey’s multiple comparison test. n = 3–7. (Abbreviations: glucose-6-phosphate (G-6-P), fructose-6-phosphate (F-6-P), 1,3-diphosphoglycerate (1,3-DPG), 3-phosphoglycerate (3-PG), 2-phosphoglycerate (2-PG), phosphoenolpyruvate (PEP), glyceraldehyde-3-phosphate (Gly-3-P), D-sedoheptulose-1,7-phosphate (D-sedoheptulose-1,7-P), tricarboxylic acid (TCA)).

In the glycolytic pathway, we found a number of metabolites that exhibited significantly different expression between HKCs and T1DMs. Fructose-1,6-bisphosphate was significantly downregulated in T1DM compared to controls (2.4-fold, p ≤ 0.01) and HKCs, as well as T2DM (p ≤ 0.01) (Fig. 3C). Of interest, while HKCs showed increased levels of glycolytic intermediates, including 1,3-diphosphoglycerate, glyceraldehyde-3-phosphate, and dihydroxyacetone phosphate, T1DM constructs displayed a downregulation of these metabolites compared to HCFs (Fig. 3C,D,F,G). Phosphoenolpyruvate was the only glycolytic metabolite upregulated in both HKCs and T1DMs compared to controls (p ≤ 0.05, Fig. 3E). The TCA cycle exhibited significantly increased expression in T1DMs compared to HKCs when analyzing expression of the metabolites malate and fumarate (p ≤ 0.01, Fig. 3H,N).

### Metabolic Differences between HKC and T2DM

Six metabolites were significantly different among the HKCs and T2DM. Two of the metabolites, 1,3-disphosphoglycerate and phosphoenolpyruvate are components of the glycolytic pathway and are contiguous in nature, as seen in Fig. 3. When compared to HKCs, T2DMs exhibit increased expression by 5-fold (p ≤ 0.001) and 1.7-fold (p ≤ 0.05) in both metabolites, respectively (Fig. 3D,E).

Interestingly, lactate and citrate were both significantly downregulated in T2DMs, when compared to HKCs.

## Discussion

KC is the most common ectatic corneal disease, which impairs vision by causing corneal thinning, bulging and in more advanced cases, corneal scarring. Clinical findings include discomfort, visual disturbances, a negative impact on everyday tasks and possible blindness if left untreated. According to Global Consensus of Keratoconus and Ectatic Diseases, 83.3% of ophthalmic physicians are performing CXL as a KC treatment. CXL is known to alter the corneal stroma ECM and strengthen the collagen bonds, leading to reduced interfibrillar spacing of collagen fibrils and increased fibril diameter^31^, ultimately increasing stiffness and arresting the disease progression.

The prolonged hyperglycemic states that the human cornea experiences during DM also lead to endogenous CXL within the corneal stroma. In the case of DM, however, the process happens naturally with age through a cascade of intramolecular and intermolecular mechanisms primarily mediated by crosslinking of advanced glycation end products (AGEs) that are produced during chronic hyperglycemia^3^. Furthermore, studies have suggested that riboflavin-mediated CXL may also proceed via *in situ* production of AGEs from endogenous glycosaminoglycans (GAGs) present on core proteoglycan proteins^32^. A few case studies have suggested that there may be a protective effect of diabetes on the development of KC. With both KC and DM shown to possess metabolic pathology, we have looked further into finding a relationship in the metabolic network of the two diseases in order to determine if this could be important into the hypothesized protective effect of DM on KC.

The actual number of studies reported are few in number and have been conflicting. The original postulate was derived from the biomechanical differences that the two diseases possessed, where the authors concluded that DM stiffens while KC weakens the human cornea^7^. From a retrospective case-control study, Seiler *et al*. determined that DM had a statistically significant protective effect against KC. Another study reported a lower prevalence of KC diagnosis in DM patients when compared to a control population^6^. In contrast, Kuo and co-authors observed no differences in prevalence among diseased and control populations but did find a negative association between DM presence and KC severity^33^. A conflicting report came from Kosker *et al*. in 2014 where the authors noted a positive association in both prevalence and severity between KC and DM^34^. These studies highlight the complexity of the potential interplay between KC and DM in the context of human corneal microenvironment.

Numerous studies have associated KC with increased oxidative stress *in vitro*^16,35^ and *ex vivo*^14,36^. Recent work has further suggested that alterations in levels of reactive oxygen species (ROS)-scavenging enzymes, such as superoxide dismutase, may contribute to defects in responding to endogenous and exogenous oxidative species by HKCs^37^. Moreover, the metabolic changes observed in our study, including an upregulation in aerobic glycolysis in HKCs characterized by increased lactate production, may correlate to altered ROS levels that in turn affects glucose metabolism^20,21^, though this trend appears absent or inversely regulated in T1DM and T2DM constructs. Though oxidative stress has been posited to play a fundamental role in both KC and DM^38–40^, we hypothesize that the metabolic phenotype found in HKCs may correspond to an inherent defect in metabolic regulation compared to the combined phenotypic and epigenotypic changes that occur following prolonged exposure to elevated glucose in the case of DM. Given that the abundance of glycolytic metabolites in T2DMs remains similar to control levels, our results suggest that the effects of DM on promoting CXL *in situ* may be more related to exogenous glucose levels than cytosolic flux. Thus, the fundamental differences in disease causation between KC and DM may partially explain the inverse regulation in basal cellular metabolism observed *in vitro* with HKCs exhibiting increased glycolytic metabolite levels compared to T1DMs. Variations between T1DM and T2DM were also evident in this study with higher expression of select metabolites in T2DM, including dimethyl-L-arginine and guanosine monophosphate; however, glycolytic metabolism appeared relatively consistent (excluding a single metabolite, fructose-6-phosphate, which was significantly higher in T1DM than T2DM). Our lab^22,23^ has previously identified significant variations between ECM protein expression by corneal fibroblasts and corneal tissue in T1DM and T2DM. While our current study did not assess relative HbA1c levels in DM patients prior to tissue isolation, it likely that chronic glycemic levels and the degree of blood glucose control impact epigenetic markers of each cell type, thus contributing to differential cell phenotype detected *in vitro*.

Of importance, the metabolomics studies evaluated in this retrospective analysis were performed in euglycemic media, thus, further investigation of the differential responses of T1DMs and T2DMs challenged with a high glucose environment is justified. Furthermore, though a report suggesting that acute exposure to elevated glucose alone may not promote altered keratocyte marker expression, *e.g*. keratocan and lumican, by healthy corneal stromal stem cells *in vitro*^41^, stimulation with chronic hyperglycemia may promote a differential response in corneal fibroblasts isolated from DM patients due to permanent mitochondrial damage and increased ECM thickness^22^. We posit that the epigenetic changes that occur during prolonged DM^42,43^ may give rise to the characteristic defects in corneal structure, including differences in ECM composition^22,23^. Further studies are required to elucidate the connection between altered collagen isoform secretion favoring a fibrotic phenotype characterized by increased Collagen III and changes in glucose metabolism in HKCs^16,21^ and DM^22,23^, *i.e*. aerobic glycolysis versus TCA and the pentose phosphate pathway. These pathways may also play a significant role in the response to hyperglycemia by T1DMs and T2DMs. The mechanism by which this pathological change occurs during DM and more importantly, if this process can be reversed with pharmacological intervention, remains a significant question. Our study is the first to our knowledge to shed light on the metabolic similarities and differences of KC and DM to better define the observed biomechanical alterations that occur in the cornea.

## Data Availability

The datasets generated during the current study are available from the corresponding author on reasonable request.
